# Rapid Detection of Carbapenemase-producing *Enterobacteriaceae*

**DOI:** 10.3201/eid1809.120355

**Published:** 2012-09

**Authors:** Patrice Nordmann, Laurent Poirel, Laurent Dortet

**Affiliations:** Faculté de Médecine Paris Sud, Le Kremlin-Bicêtre, France (P. Nordmann, L. Poirel, L. Dortet);; and Institut National de la Santé et de la Recherche Médicale, Paris, France (P. Nordmann, L. Poirel, L. Dortet)

**Keywords:** antimicrobial drug resistance, antibiotic, gram-negatives bacteria, β-lactamase, carbapenemase, *Enterobacteriaceae*

## Abstract

To rapidly identify carbapenemase producers in *Enterobacteriaceae*, we developed the Carba NP test. The test uses isolated bacterial colonies and is based on in vitro hydrolysis of a carbapenem, imipenem. It was 100% sensitive and specific compared with molecular-based techniques. This rapid (<2 hours), inexpensive technique may be implemented in any laboratory.

Multidrug resistance is emerging worldwide at an alarming rate among a variety of bacterial species, causing both community-acquired and nosocomial infections (*1*). Carbapenems, the last line of therapy, are now frequently needed to treat nosocomial infections, and increasing resistance to this class of β-lactams leaves the health care system with almost no effective drugs (*1*). However, reports of carbapenem-resistant *Enterobacteriaceae* have increased (*2*,*3*). Resistance may be related to association of a decrease in bacterial outer-membrane permeability, with overexpression of β-lactamases with no carbapenemase activity or to expression of carbapenemases (*2*,*4*,*5*). Spread of carbapenemase producers is a relevant clinical issue because carbapenemases confer resistance to most β-lactams (*2*). Various carbapenemases have been reported in *Enterobacteriaceae,* such as the following types: *Klebsiella pneumoniae* carbapenemase (KPC; Ambler class A); Verona integron–encoded metallo-β-lactamase (VIM), imipenemase (IMP), New Delhi metallo-β-lactamase (NDM) (all Ambler class B); and oxacillinase-48 (OXA-48; Ambler class D) (*2*,*4*–*6*). In addition, carbapenemase producers are usually associated with many other non–β-lactam resistance determinants, which give rise to multidrug- and pandrug-resistant isolates (*2*,*3*,*7*).

Potential carbapenemase producers are currently screened first by susceptibility testing, using breakpoint values for carbapenems (*2*,*8*). However, this technique is time-consuming, and many carbapenemase producers do not confer obvious resistance levels to carbapenems. There is a need for laboratories to search for carbapenemase producers (*9*). Phenotype-based techniques for identifying in vitro production of carbapenemase, such as the modified Hodge test, are not highly sensitive and specific (*2*,*8*,*10*). Detection of metallo-β-lactamase producers (IMP, VIM, NDM) and of KPC producers may be based on the inhibitory properties of several molecules but requires additional expertise and time (usually an extra 24–48 hours) (*2*,*8*,*11*,*12*). Furthermore, no inhibitors are available for detecting OXA-48–type producers that are spreading rapidly, at least in northern Africa, the Middle East, and Europe (*2*). Molecular detection of carbapenemase genes remains costly and requires substantial expertise. Both the phenotype-based techniques and molecular tests are time-consuming (at least 12–24 hours) and are poorly adapted to the clinical need for isolating patients rapidly to prevent nosocomial outbreaks.

We developed a novel test, described here, based on a technique designed to identify the hydrolysis of the β-lactam ring of a carbapenem. This test is rapid, sensitive and specific, and adaptable to any laboratory in the world.

## The Study

We included in the study 162 carbapenemase-producing strains of various enterobacterial species isolated from clinical samples (e.g., blood cultures, urine, sputum) and of global origin (Table 1). This collection of strains also included 46 strains that were fully susceptible to carbapenems or showed a decreased susceptibility to carbapenems as a consequence of non–carbapenemase-based mechanisms (Table 2). Antibiograms were carried out for all strains on Mueller-Hinton agar (Becton Dickinson, Le Point de Chaix, France) according to guidelines of the Clinical and Laboratory Standards Institute (*13*). The Carba NP (Carbapenemase Nordmann-Poirel) test was performed as follows. One calibrated loop (10 μL) of the tested strain directly recovered from the antibiogram was resuspended in a Tris-HCl 20 mmol/L lysis buffer (B-PERII, Bacterial Protein Extraction Reagent; Thermo Scientific Pierce, Rockford, IL, USA), vortexed for 1 minute and further incubated at room temperature for 30 minutes. This bacterial suspension was centrifuged at 10,000 × *g* at room temperature for 5 minutes. Thirty µL of the supernatant, corresponding to the enzymatic bacterial suspension, was mixed in a 96-well tray with 100 µL of a 1-mL solution made of 3 mg of imipenem monohydrate (Sigma, Saint-Quentin Fallavier, France), pH 7.8, phenol red solution, and 0.1 mmol/L ZnSO_4_ (Merck Millipore, Guyancourt, France). The phenol red solution was prepared by mixing 2 mL of a phenol red (Merck Millipore) solution 0.5% (wt/vol) with 16.6 mL of distilled water. The pH value was then adjusted to 7.8 by adding drops of 1 N NaOH. A mixture of the phenol red solution and the enzymatic suspension being tested was incubated at 37°C for a maximum of 2 hours. Test results were interpreted by technicians who were blinded to the identity of the patients who gave the samples.

**Table 1:** Carbapenemase-producing clinical enterobacterial isolates subjected to the Carba NP test*

Ambler class, carbapenemase type	Species	β-Lactamase	No.	MIC range, mg/L			Carba NP test result
				IMP	ERT	MER	
Class A							
KPC-type	*Klebsiella pneumoniae*	KPC-2	27	0.5–>32	4–>32	1–>32	+
		KPC-3	3	0.5–8	4–>32	1–8	+
	*Klebsiella ozaenae*	KPC-3	1	>32	>32	2	+
	*Escherichia coli*	KPC-2	5	0.5–4	0.5>32	0.5–2	+
	*Enterobacter cloacae*	KPC-2	7	1–24	1.5–32	0.75–16	+
	*Enterobacter aerogenes*	KPC-2	1	8	>32	8	+
	*Citrobacter freundii*	KPC-2	2	8–>32	1.5–>32	1.5–3	+
	*Serratia marcescens*	KPC-2	2	>32	>32	>32	+
	*Salmonella* spp.	KPC-2	1	4	1	0.25	+
NMC-A	*E. cloacae*	NMC-A	1	16	>32	16	+
SME-type	*S. marcescens*	SME-1	1	32	4	12	+
		SME-2	1	32	4	12	+
GES-type	*E. cloacae*	GES-5	1	>32	>32	>32	+
IMI-type	*Enterobacter asburiae*	IMI-2	1	>32	>32	>32	+
Class B							
NDM-type	*K. pneumoniae*	NDM-1	16	0.5–>32	2–>32	1–>32	+
		NDM-4	1	>32	>32	>32	+
	*E. coli*	NDM-1	7	1–16	3–>32	1–16	+
	*E. cloacae*	NDM-1	1	2	16	2	+
	*C. freundii*	NDM-1	1	>32	>32	>32	+
	*Providencia stuartii*	NDM-1	1	12	0.38	1.5	+
	*Proteus rettgeri*	NDM-1	1	3	0.5	1.5	+
VIM-type	*K. pneumoniae*	VIM-1	15	0.5–>32	0.5–>32	0.38–>32	+
		VIM-19	1	8	16	4	+
	*E. coli*	VIM-1	2	1.5–3	0.38–1.5	0.5–1	+
		VIM-2	2	2–4	0.5–1.5	0.38–0.5	+
		VIM-19	1	8	16	4	+
	*E. cloacae*	VIM-1	4	1–>32	0.38 to >32	0.5–>32	+
	*S. marcescens*	VIM-2	1	>32	>32	>32	+
IMP-type	*K. pneumoniae*	IMP-1	5	0.5–8	2–4	1–8	+
		IMP-8	2	0.5–1	0.5–1	0.5	+
	*E. coli*	IMP-1	2	0.5	3–4	0.5–1	+
		IMP-8	1	6	8	3	+
	*E. cloacae*	IMP-1	12	8–>32	>32	2–>32	+
		IMP-8	2	0.75–1.5	0.5–1	0.5–1	+
	*S. marcescens*	IMP-1	2	8–>32	>32	2–>32	+
		IMP-11	1	8	>32	2	+
Class D							
OXA-48 type	*K. pneumoniae*	OXA-48	15	0.38–>32	0.38–>32	0.38–>32	+
		OXA-181	2	0.5-1	2–4	0.5–1	+
	*E. coli*	OXA-48	6	0.38–3	0.5–16	0.12–1	+
	*E. cloacae*	OXA-48	3	0.5–1	0.5–16	0.5–1.5	+
	*P. rettgeri*	OXA-181	1	8	1	2	+

*IMP, imipenem; ERT, ertapenem; MER, meropenem; KPC, *K. pneumoniae* carbapenemase; NMC-A, non–metallo-enzyme carbapenemase; SME, *S. marscescens* enzyme; GES, Guiana extended-spectrum β-lactamase; IMI, imipenem-hydrolysing β-lactamase; NDM, New Delhi metallo-β-lactamase; VIM, Verona integron–encoded metallo-β-lactamase; IMP, imipenemase; OXA-48, oxacillinase-48.

**Table 2:** Non–carbapenemase-producing clinical enterobacterial isolates subjected to the Carba NP test*

β-Lactamase type, species	β-Lactamase	No.	MIC, mg/L			Carba NP test result
			IMP	ERT	MER	
ESBLs						
* Klebsiella pneumoniae*	CTX-M-3	1	0.12	0.12	0.12	–
	CTX-M-14	1	0.12	0.12	0.12	–
	CTX-M-15	3	0.12	0.12	0.12	–
* Escherichia coli*	CTX-M-1	1	0.12	0.12	0.12	–
	CTX-M-3	1	0.12	0.12	0.12	–
	CTX-M-14	2	0.12	0.12	0.12	–
	CTX-M-15	2	0.12	0.12	0.12	–
	VEB-1	1	0.12–0.25	0.12	0.12	–
* Enterobacter cloacae*	CTX-M-15	3	0.12	0.12	0.12	–
	VEB-1	1	0.12	0.12	0.12	–
Plasmid-mediated AmpC or chromosomal AmpC + decreased membrane permeability						
* K. pneumoniae*	DHA-1	1	>32	>32	>32	–
	DHA-2	1	0.12	0.5	0.12	–
* E. coli*	Extended-spectrum cephalosporinase	1	0.12	0.12	0.12	–
	CMY-2	1	0.12	0.12	0.12	–
	CMY-10	1	0.12	0.38	0.12	–
	DHA-1	1	0.12	0.12	0.12	–
	ACC-1	1	0.12	0.12	0.12	–
	Overexpressed cephalosporinase	1	16	>32	2	–
* Proteus mirabilis*	ACC-1	1	0.25	0.12	0.12	–
* E. cloacae*	Overexpressed cephalosporinase	7	0.12–16	1–>32	0.12–>32	–
* Enterobacter aerogenes*	Overexpressed cephalosporinase	1	1	4	0.75	–
* Morganella morganii*	Overexpressed cephalosporinase	2	1.5–2	0.12	0.5	–
ESBL + decreased membrane permeability						
* K. pneumoniae*	CTX-M-15	8	0.25–8	1–>32	1–>32	–
	SHV-28	1	1	4	1	–
	SHV-2a	1	0.25	2	0.38	–
* Enterobacter sakazakii*	CTX-M-15	1	0.25	1.5	0.25	–
* Citrobacter freundii*	TEM-3	1	1	8	1	–

*IMP, imipenem; ERT, ertapenem; MER, meropenem; ESBLs, extended-spectrum β-lactamases.

All strains had previously been characterized for their β-lactamase content at the molecular level. MICs of carbapenems were determined by using the Etest (AB bioMérieux, Solna, Sweden), and results were recorded according to US guidelines (Clinical and Laboratory Standards Institute), as updated in 2012 (*13*). The breakpoints used were those for imipenem and meropenem: susceptibility <1 μg/mL, resistance >4 µg/mL, and for ertapenem, susceptibility <0.25 μg/mL, resistance > µg/mL.

**Figure 1:**
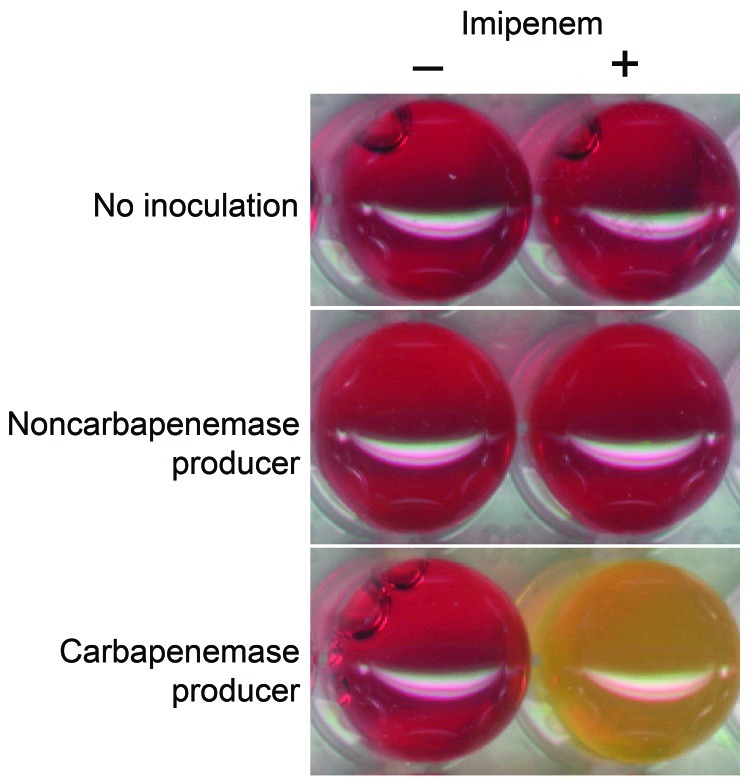
Representative results of the Carba NP test. The Carba NP test was performed with a noncarbapenemase producer (*Escherichia coli* producing the extended-spectrum β-lactamase CTX-M-15, upper panel) and with a carbapenemase producer (*Klebsiella pneumoniae*–producing New Delhi metallo-β-lactamase-1, lower panel) in a reaction medium without (left panel) and with (right panel) imipenem. Uninoculated wells are shown as controls. Photographs were taken after a 1.5-hour incubation.

When the Carba NP test was used, the color of the wells turned from red to orange or yellow (Figure 1) for all tested strains that were producing carbapenemases (Table 1), whereas wells corresponding to bacterial extracts of isolates that did not produce carbapenemase remained red, whatever their level of carbapenem susceptibility (Table 2). The color changed from red to yellow as early as 5–10 minutes after incubation for KPC producers began. In most cases, incubation for 30 minutes was sufficient for obtaining a frank color change for carbapenemase producers. The test’s specificity and sensitivity were 100% when results were compared with those from molecular-based methods, the reference standard for identifying carbapenemase genes. All tests were performed in triplicate, giving identical and reproducible results.

The Carba NP test perfectly differentiates carbapenemase producers (Table 1) from strains that are carbapenem resistant due to non–carbapenemase-mediated mechanisms, such as combined mechanisms of resistance (outer-membrane permeability defect associated with overproduction of cephalosporinase and/or extended-spectrum β-lactamases) or from strains that are carbapenem susceptible but express a broad-spectrum β-lactamase without carbapenemase activity (extended-spectrum β-lactamases, plasmid and chromosome-encoded cephalosporinases) (Table 2). Interpretable positive results were obtained in <2 hours, making it possible to implement rapid containment measures to limit the spread of carbapenemase producers.

## Conclusions

The Carba NP test has multiple benefits. It is inexpensive, rapid, reproducible, and highly sensitive and specific. It eliminates the need for using other techniques to identify carbapenemase producers that are time-consuming and less sensitive or specific. Using this accurate test would improve detection of patients infected or colonized with carbapenemase producers. The test has been routinely implemented in our microbiology department at Hôpital de Bicêtre and is giving excellent results (data not shown). In addition, use of the Carba NP test has greatly decreased the laboratory technicians’ workload and simplified the clinical management of potential carbapenemase producers.

**Figure 2:**
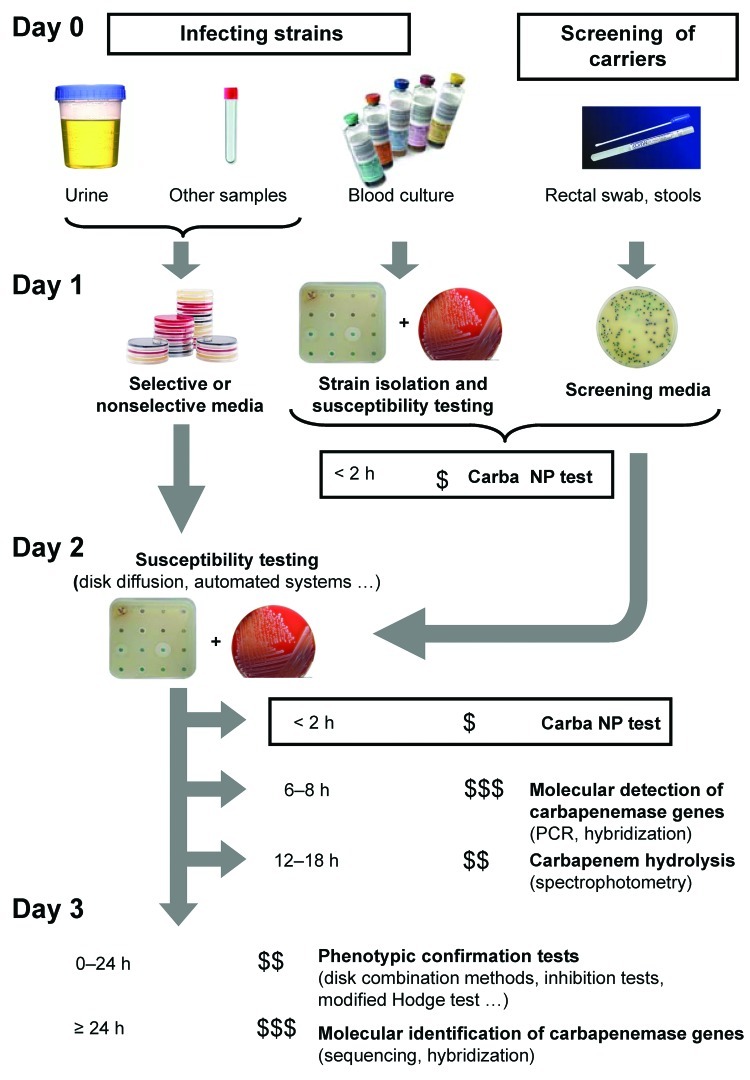
Strategy for identification of carbapenemase-producing *Enterobacteriaceae*. The time needed to perform the test is indicated before each test. The number of flasks indicates the degree of specialization needed to perform the test; the number of $ indicates the relative cost of each test.

This test could be used, for example, for directly testing 1) bacteria obtained from antibiograms of blood culture or 2) bacterial colonies grown on culture media before antimicrobial drug susceptibility testing (Figure 2). Further studies will evaluate its clinical value for antimicrobial drug stewardship on bacteria isolated directly from clinical samples (Figure 2). When the Carba NP test is used for that purpose, we expect that the time to detect carbapenemase producers will decrease by at least 24 hours (Figure 2).

The test could also be used to quickly identify carbapenem-resistant isolates from fecal specimens screened for multidrug-resistant bacteria (Figure 2). This capability would be valuable in preventing outbreaks. To determine positive and negative predictive values of the test, additional evaluations will be required with strains isolated from clinical samples screened on different types of selective media. The use of the Carba NP test may also support novel antimicrobial drug development by facilitating patient enrollment in pivotal clinical trials. Its use as a home-made test may contribute to the global surveillance network.

The Carba NP test can efficiently indicate the strains to be further tested by PCR or submitted to sequencing for a detailed identification of the carbapenemase genes. Last, the test could be used in low-income countries that are large reservoirs for carbapenemase producers (*2*). It offers a practical solution for detecting a main component of multidrug resistance in *Enterobacteriaceae*. Use of the Carba NP test will contribute to a better stewardship of carbapenems by changing the paradigm of controlling carbapenemase producers worldwide
